# Alterations in neuroplasticity and functional connectivity of striatal subregions in Bell’s palsy patients after acupuncture

**DOI:** 10.3389/fneur.2025.1684824

**Published:** 2026-01-12

**Authors:** Zelin Yu, Wenwen Song, Binyan Yu, Yena Gu, Minhui Dai, Maosheng Xu, Lihua Xuan

**Affiliations:** Zhejiang Chinese Medical University, Hangzhou, Zhejiang, China

**Keywords:** acupuncture, Bell’s palsy, fMRI, functional connectivity, striatum

## Abstract

**Background:**

Bell’s palsy (BP) is an acute facial palsy caused by the inflammation of the facial nerve. Previous research indicates that the striatum may be involved following acute peripheral nerve injury, and acupuncture is a recognized treatment for BP. However, it remains unclear whether the striatum is functionally engaged during the recovery process with acupuncture.

**Method:**

Using resting-state functional MRI (fMRI), we investigated striatum-related neural activity in BP patients by measuring two key metrics of local brain function: regional homogeneity (ReHo, reflecting local neural synchrony) and fractional amplitude of low-frequency fluctuations (fALFFs, reflecting the intensity of spontaneous neural activity). We further examined corticostriatal and internal striatal functional connectivity. Patients underwent fMRI scans before and immediately after (15 min following needle withdrawal) an acupuncture treatment session to capture dynamic changes.

**Results:**

The post-treatment scan was associated with significant alterations in both ReHo and fALFFs, including increased fALFFs in the left postcentral gyrus and the precentral gyrus and increased ReHo in the right cerebellum (Crus2). Several striatal subregions also exhibited significantly enhanced internal connectivity.

**Conclusion:**

Our results indicate that the striatum undergoes functional alterations during the recovery period, which may provide preliminary insight into neural processes associated with treatment for BP.

## 1 Introduction

Bell’s palsy (BP) is an acute unilateral facial paralysis caused by inflammation of the facial nerve (1–3). Acupuncture is widely used as an effective treatment for BP, and the World Health Organization has listed BP among the recommended indications for acupuncture since 1996 (4). However, despite its clinical application, the underlying mechanisms through which acupuncture contributes to facial nerve recovery in BP remain unclear (5–7).

Previous studies by our group (8, 9) demonstrated that the striatum is affected following BP at the acute stage and suggest that this disrupted striatal functional connectivity may reflect a compensatory mechanism for the sensory-motor mismatch caused by BP.

The human basal ganglia are fundamental to integrated brain function. The striatum, which consists of the caudate nucleus and the lentiform nucleus, is the principal input structure of the basal ganglia (10). The striatum can accept nerve projections from both the cortex and the thalamus and then project back to the cortex after channeling through pallidal and thalamic nuclei, which is called the corticostriatal “loops” (11, 12). Some motor dysfunctions, such as Parkinson’s disease and Huntington’s disease, affect functional connectivity between the striatum and the motor cortex. Blood et al. (13) found that connectivity among striatal subdivisions decreases, while connectivity between the striatum and other cerebral regions increases, in X-linked dystonia-parkinsonism. Bell et al. (14) found that administration of dopaminergic medication significantly improved connectivity across striatal subdivisions in Parkinson’s disease.

In this study, we first used resting-state fMRI to compare regional brain functional status in the acute stage and after acupuncture using ReHo and fALFFs. We then compared whole-brain rsFC changes using 12 striatal subregions as ROIs and examined rsFC among each of the 12 striatal seeds. Our objective was to explore changes in the striatum across the acupuncture treatment period. It remains unclear whether and how acupuncture is associated with striatal function in restoring the injured facial nerve. We hypothesized that acupuncture would change abnormal functional connectivity between the striatum and other brain regions caused by acute peripheral nerve injury. Moreover, we aimed to explore whether acupuncture stimulation induces changes in the ipsilateral or contralateral striatum.

## 2 Materials and methods

### 2.1 Participants and clinical assessment

Thirty patients with BP were enrolled at our hospital from June 2020 to December 2021. BP was diagnosed by two experienced physicians using the House–Brackmann (H–B) score. All patients had a disease course of Bell’s palsy of less than 14 days and had not received any treatment. Their H–B scores were IV ~ VI. No participants had a history of physical or mental disorders. Participants were not compensated for study participation. The study was approved by the Ethics Committee of the First Affiliated Hospital of Zhejiang Chinese Medical University and registered with the Chinese Clinical Trial Registry (ChiCTR-INR-16008410). Table 1 shows the clinical information.

**Table 1:** Clinical data for participants.

Clinical score		Pre-treatment (*n* = 20)	Post-treatment (*n* = 20)	*Z*	*p*
HB score	I	0	2		
	II	0	6		
	III	0	3	−3.88	<0.01
	IV	1	3		
	V	13	5		
	VI	6	1		

The Wilcoxon test was used.

### 2.2 Interventions

Disposable acupuncture needles (size 0.25*40 mm; Jaichen, Jianshu, China) were used. Participants received acupuncture at ipsilateral Yangbai (GB14), Yuyao (EX-HN4), Taiyang (EX-HN5), Cuanzhu (BL2), Yingxiang (LI20), Jiache (ST6), Xiaguan (ST7), Dicang (ST4), Quanliao (SI18), and bilateral Hegu (LI4). Participants received 4 treatment sessions per week for 4 consecutive weeks, 16 sessions in total. Participants underwent the initial MRI scan immediately after enrollment. The second MRI scan was performed on the last day of the 4-week treatment, 15 min following withdrawal of needles.

All acupuncture procedures were performed by a senior chief physician with over 30 years of clinical experience specializing in the treatment of facial palsy.

For needle insertion, GB14, EX-HN4, and EX-HN5 were inserted horizontally, whereas BL2, LI20, ST6, ST7, ST4, SI18, and LI4 were inserted perpendicularly according to standard clinical practice.

After insertion at bilateral LI4, manual stimulation (lifting-thrusting and twirling) was applied to elicit the characteristic “deqi” sensation, typically described by patients as numbness, distention, or heaviness. Needle manipulation was standardized across sessions to ensure consistency of stimulation intensity.

### 2.3 fMRI imaging acquisition

MRI scans were performed with a 3.0 Tesla MR scanner (Magnetom Verio, Siemens, Germany) to obtain T1-weighted structural images and echo-planar T2*-weighted images (EPIs). Structural images were acquired using the MP-RAGE sequence: TR = 1900 ms, TE = 2.45 ms, FA = 9 °, voxel size = 1 × 1 × 1 mm, matrix = 256 × 256. Two hundred time points of functional resting state data were acquired using an EPI session: TR = 2000 ms, TE = 30 ms, FA = 90°, slices = 33, voxel size = 4.0 × 4.0 × 4.0 mm, matrix = 256 × 256.

### 2.4 fMRI data preprocessing

Since the included BP patients had unilateral facial palsy on either the left or the right side, we applied a right–left (R–L) flipping procedure to all images of left-sided palsy to ensure that the affected side was aligned across participants. This procedure was implemented by mirroring the individual functional images along the y-axis prior to group-level analysis.

Importantly, following established procedures in prior neuroimaging studies of lateralized motor function (15), this flipping step does not equate the left and right hemispheres. Instead, it allows us to treat the “affected side” consistently across participants while preserving hemispheric asymmetries at the group level. All images underwent standard spatial normalization after flipping, ensuring that the procedure did not distort anatomical alignment. To confirm this, we visually inspected all normalized images and confirmed that no systematic misregistration or deformation occurred as a result of the flipping process.

Functional MRI data analysis was performed using a seed-based approach with the CONN toolbox v15.g (16).^1^ The preprocessing of fMRI data was performed using Statistical Parametric Mapping (SPM8) (Welcome Department of Cognitive Neurology, University College, London, UK) in MATLAB (MathWorks, Inc., Natick, MA, USA; (17)). The preprocessing steps included realignment, coregistration of subjects’ respective functional and structural images, normalization, and smoothing with an 8-mm full-width at half maximum (FWHM) kernel. In addition to these steps, we used segmentation of gray matter, white matter, and cerebrospinal fluid (CSF) areas in order to remove temporal confounding factors (16). Band-pass filtering was performed in a frequency window of 0.008–0.09 Hz. Individual regional homogeneity (ReHo) maps were computed on the filtered data to assess local neural synchrony, while the fractional amplitude of low-frequency fluctuation (fALFF) maps were calculated directly on preprocessed data without band-pass filtering to quantify the relative power of spontaneous low-frequency (0.01–0.08 Hz) oscillations.

### 2.5 Functional resting state connectivity analysis

We used six striatum subregions in each hemisphere as 3 mm spherical seeds. The centers of the seeds were dorsal caudal putamen (DCP, NMI coordinate, ±28, 1, 3), ventral rostral putamen (VRP, ±20, 12, −3), dorsal rostral putamen (DRP, ±25, 8, 6), inferior ventral striatum (VSi, ±9, 9, −8), dorsal caudate (DC, ±13, 15, 9), and superior ventral striatum (VSs, ±10, 15, 0) (18). Seeds were selected using WFU-Pick Atlas software (19) according to NMI coordinates. Multiple comparisons were corrected using the Gaussian random field (GRF) theory with a voxel-level threshold of *p* < 0.001 and a cluster-level threshold of *p* < 0.05. For the 12*12 seed-to-seed functional connectivity analysis, multiple comparisons across all pairwise connections were corrected using the false discovery rate (FDR, *q* < 0.05).

Significant results and corresponding brain regions were visualized with BrainNet Viewer (Version 1.7).

### 2.6 Correlation analysis between FC and clinical scores

To further evaluate the clinical relevance of the neuroimaging findings, we performed correlation analyses between significant functional connectivity (FC) alterations and House–Brackmann (HB) scores. For each subject, FC values were extracted from brain regions that showed significant changes in the group-level FC analysis using SPM. The extracted FC values were then correlated with corresponding HB scores using Spearman’s rank correlation, given the ordinal nature of the HB scale. All analyses were conducted using SPSS (Version 26) and MATLAB R2017, with statistical significance defined as a *p*-value of < 0.05 (two-tailed).

## 3 Results

### 3.1 Patient characteristics

A total of 20 patients completed this trial. Five patients did not complete the second scan, and another five were excluded due to head motion exceeding 3 mm. The pre-treatment and post-treatment HB scores are shown in Table 1. There was a significant difference between the pre-treatment and post-treatment regarding the HB score (*p* < 0.01).

### 3.2 ReHo and fALFF results between pre-treatment and post-treatment

**Figure 1:**
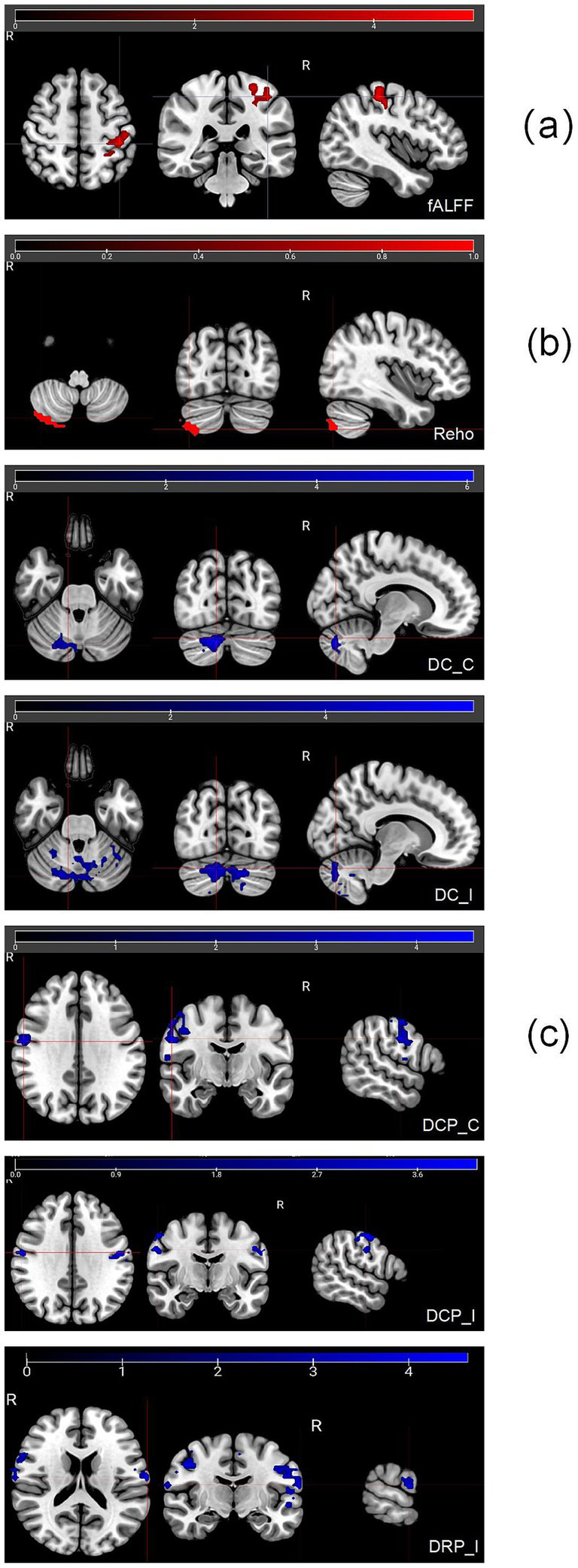
Resting-state fMRI results. GRF corrected with a voxel-level threshold of *p* < 0.001 and a cluster-level threshold of *p* < 0.05 **(a)** Post-treatment results showed significant fALFF increases in the contralateral postcentral gyrus and precentral gyrus. The highlighted region shows the postcentral gyrus and precentral gyrus. **(b)** Post-treatment results showed significant ReHo increases in the ipsilateral cerebellum Crus2. The highlighted region shows the cerebrum. **(c)** Functional connectivity results based on twelve striatum seeds. The red point indicates the locations of seeds in the brain.

At 4 weeks post-treatment, the results showed (1) significant increases in fALFFs in the contralateral postcentral gyrus and the precentral gyrus and (2) significant ReHo increases in the ipsilateral cerebellum Crus2 (Table 2; Figures 1a,b).

**Table 2:** Results of resting-state fMRI.

Resting-state	Contrast	Brain region	Cluster size	Peak Z score	MNI coordinates (mm)		
					x	y	z
fALFFs	Post>Pre	Postcentral_L(aaL)	239	4.21	−39	−27	60
		Precentral Gyrus_L	93				
ReHo	Post>Pre	Cerebrlum_Crus2_R	135	5.09	12	−81	−33

Pre, pre-treatment; Post, post-treatment.

### 3.3 rsFC results between pre-treatment and post-treatment

Following acupuncture treatment, significant alterations in corticostriatal and internal striatal functional connectivity were observed, primarily involving the dorsal striatum. As illustrated in Figure 1c, functional connectivity between dorsal striatal seeds and cortical sensorimotor regions (precentral and postcentral gyri) and the cerebellum was significantly modulated, whereas no suprathreshold changes were detected for ventral striatal seeds.

Specifically, the post-treatment scan showed changes characterized by decreased connectivity between (i) the bilateral dorsal caudate (DC) and the ipsilateral cerebellum (Crus1); (ii) the ipsilateral dorsal caudal putamen (DCP) and the contralateral postcentral gyrus as well as the ipsilateral precentral gyrus, with the contralateral DCP showing similar decreased connectivity to the ipsilateral precentral gyrus; and (iii) the ipsilateral dorsal rostral putamen (DRP) and the bilateral postcentral gyrus, ipsilateral fusiform gyrus, and contralateral supplementary motor area (SMA). No significant connectivity changes were found for the ventral rostral putamen (VRP), inferior ventral striatum (VSi), or superior ventral striatum (VSs) (Figure 1c).

**Figure 2:**
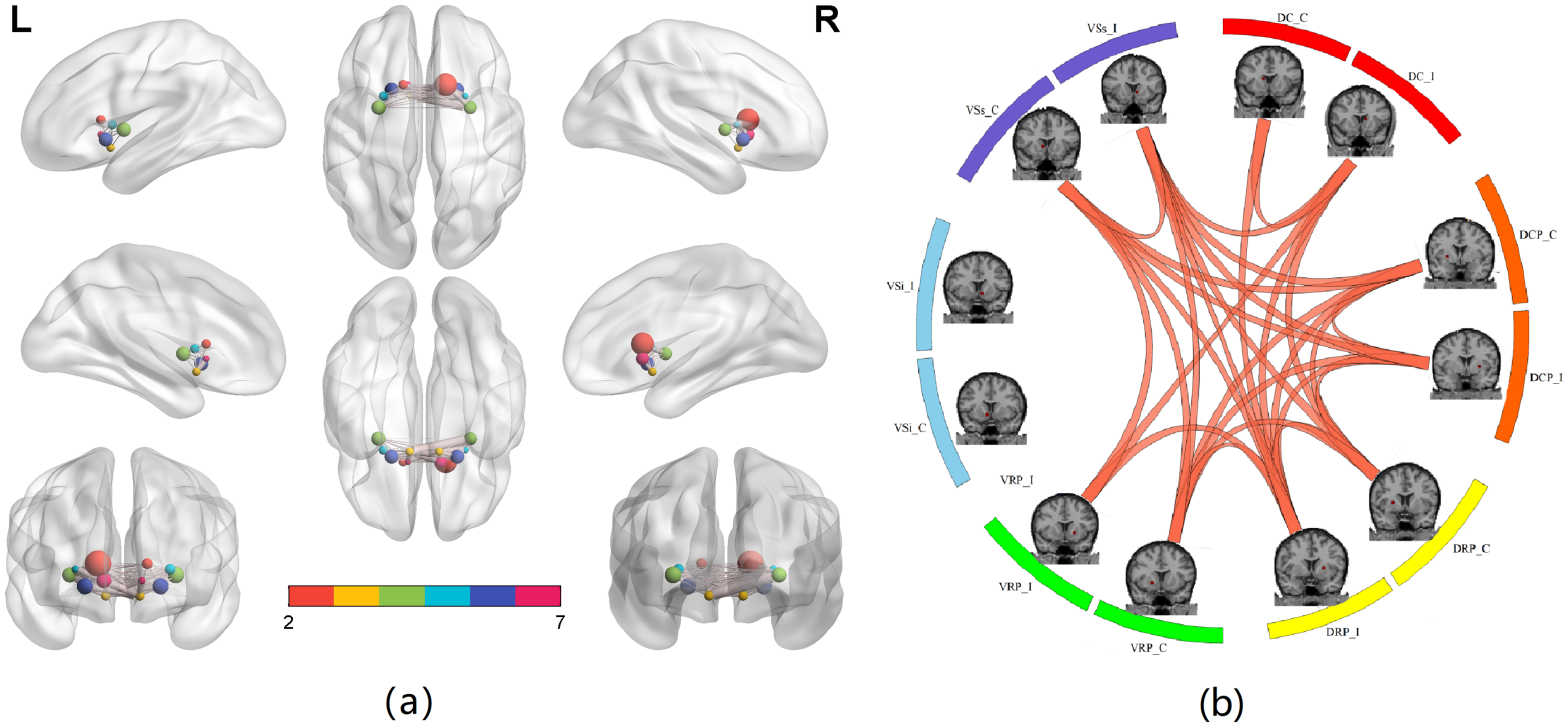
FC within the striatum. **(a)** Locations of 12 striatum subregion seeds and their FC. The size of each edge is proportional to the strength of the functional connection between seeds. The result shows that the ipsilateral DRP, VRP, and VS seeds exhibit stronger connectivity than their contralateral counterparts. **(b)** FC within the 12 striatum subregion seeds. The red points indicate the locations of seeds in the brain. The orange lines indicate enhanced connectivity between two seeds after treatment. For the 12*12 seed-to-seed functional connectivity analysis, multiple comparisons across all pairwise connections were corrected using the false discovery rate (FDR, *q* < 0.05).

Furthermore, analysis of internal striatal connectivity based on the 12 subregional seeds revealed significant reorganization within the striatal network itself, as detailed in Figure 2. The results show that the ipsilateral DRP, VRP, and VS seeds have stronger connectivity than the contralateral (see Table 3).

**Table 3:** Comparison of the connectivity in the putamen seeds.

Seeds	Brain region	Cluster size	Peak z score	MNI coordinates (mm)		
				x	y	z
Post>Pre						
DC_C	Cerebellum_Crus1_I	96	6.22	12	−75	−33
DC_I	Cerebellum_Crus1_I	246	−5.16	42	−69	−24
DCP_C	Precentral Gyrus_I	275	−4.65	57	−9	30
DCP_I	Postcentral Gyrus_C	84	−4.28	−54	−12	36
	Precentral Gyrus_I	122	−4.54	57	−3	48
DRP_C	NO					
DRP_I	Fusiform_I	261	−4.78	30	−60	−3
	Postcentral Gyrus_C	186	−4.99	−66	−12	18
	Postcentral Gyrus_I	209	−4.78	57	−9	33
	SMA_C	90	−5.34	−18	0	66
VRP_C	NO					
VRP_I	NO					
VSi_C	NO					
VSi_I	NO					
VSs_C	NO					
VSs_I	NO					

C, contralateral; I, ipsilateral; Pre: pre-treatment; Post: post-treatment.

### 3.4 Correlation analysis between FC and HB scores

**Figure 3:**
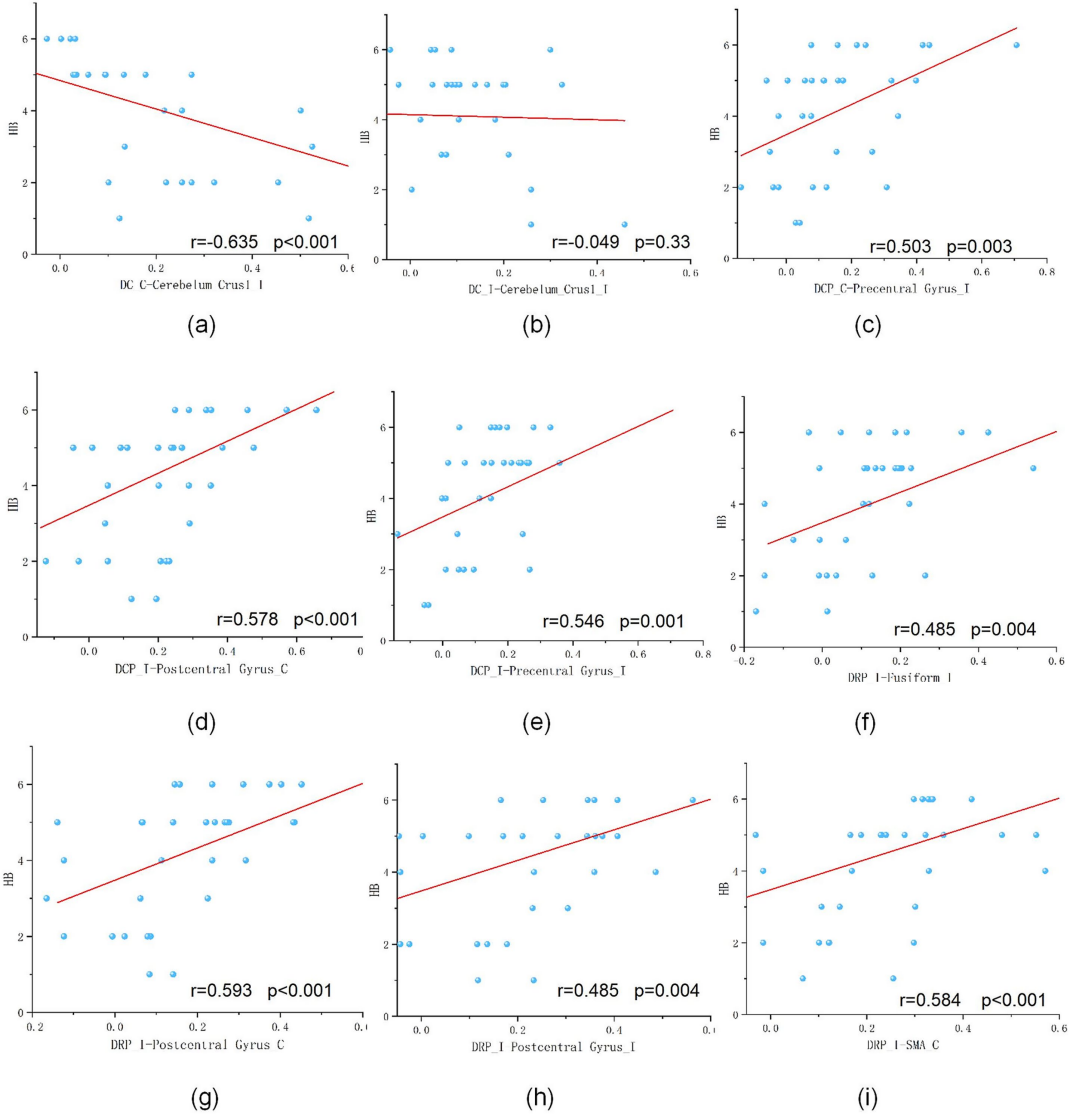
Correlation analysis between functional connectivity and HB scores. This figure illustrates the significant correlations between the functional connectivity (FC) of striatal subregions and patients’ HB scores. Positive correlations were observed for: DCP (contralateral)-Precentral gyrus (*r* = 0.503, *p* = 0.003) **(c)**; DCP (ipsilateral)-Postcentral gyrus (contralateral) (*r* = 0.578, *p* < 0.001) **(d)**; DCP (ipsilateral)-Precentral gyrus (ipsilateral) (*r* = 0.546, *p* = 0.001) **(e)**; DRP (ipsilateral)-Fusiform gyrus (ipsilateral) (*r* = 0.485, *p* = 0.004) **(f)**; DRP (ipsilateral)-Postcentral gyrus (contralateral) (*r* = 0.593, *p* < 0.001) **(g)**; DRP (ipsilateral)-Postcentral gyrus (ipsilateral) (*r* = 0.485, *p* = 0.004) **(h)**; DRP (ipsilateral)-SMA (contralateral) (*r* = 0.584, *p* < 0.001) **(i)**. A significant negative correlation was found between: DC (contralateral)-Cerebellum crus I (ipsilateral) (*r* = −0.635, *p* < 0.001) **(a)**. No significant correlation was observed for: DC (ipsilateral)-Cerebellum crus I (ipsilateral) **(b)**.

We further explored the relationship between patients’ HB scores and their brain functional connectivity (Figure 3). The results revealed several significant positive correlations:

FC between DCP (contralateral) and the precentral gyrus (*r* = 0.503, *p* = 0.003);
FC between DCP (ipsilateral) and the postcentral gyrus (contralateral) (*r* = 0.578, *p* < 0.001);
FC between DCP (ipsilateral) and the precentral gyrus (ipsilateral) (*r* = 0.546, *p* = 0.001);
FC between DRP (ipsilateral) and the fusiform gyrus (ipsilateral) (*r* = 0.485, *p* = 0.004);
FC between DRP (ipsilateral) and the postcentral gyrus (contralateral) (*r* = 0.593, *p* < 0.001);
FC between DRP (ipsilateral) and the postcentral gyrus (ipsilateral) (*r* = 0.485, *p* = 0.004); and
FC between DRP (ipsilateral) and the SMA (contralateral) (*r* = 0.584, *p* < 0.001).

In contrast, a negative correlation with HB scores was found between FC in DC (contralateral) and the cerebellum_Crus I (ipsilateral) (*r* = −0.635, *p* < 0.001).

The remaining FC between DC (ipsilateral) and cerebellum_Crus1_I (ipsilateral) showed no significant correlation.

## 4 Discussion

In the present study, we examined longitudinal neural changes in early-stage BP patients before and after acupuncture treatment. Alterations in both ReHo and fALFFs were identified, primarily involving the precentral and postcentral gyri, as well as the cerebellar regions. These areas form key components of the sensorimotor network and are frequently reported in studies of motor dysfunction and recovery.

The precentral gyrus plays a critical role in voluntary facial motor control (20, 21). Given that BP is characterized by impaired and asynchronous facial muscle movement (22), the decreased ReHo/fALFFs observed after treatment may reflect a normalization of cortical hyperactivity that typically emerges in the acute stage as part of compensatory motor recruitment. This interpretation is consistent with previous studies showing that early BP involves widespread cortical and subcortical reorganization (8, 9, 23).

Beyond cortical alterations, our findings revealed significant changes in corticostriatal connectivity, especially in the dorsal striatal regions. Most dorsal striatal seeds (DC, DCP, and DRP) showed decreased connectivity with the precentral and postcentral gyri after treatment, whereas the ventral striatal seeds demonstrated no significant whole-brain connectivity changes. Since the dorsal striatum is strongly associated with sensorimotor processing (18), these reductions in connectivity may reflect decreased compensatory sensorimotor integration demands as facial motor function improves. This interpretation is in line with previous studies showing that abnormal dorsal striatal connectivity is present during acute BP (8, 9, 24) and that striatal plasticity is important for motor recovery (25, 26).

In addition, we observed enhanced intrinsic connectivity among several striatal subregions, including bilateral DC, DCP, DRP, and VS seeds. Previous studies have indicated that the caudate contains a higher proportion of movement-related neurons than the putamen (27, 28). The increased internal connectivity within the caudate subregions may therefore represent strengthened intrastriatal coordination during the recovery process. The finding that ipsilateral DRP, VRP, and VS seeds exhibited particularly strong internal connectivity is consistent with their known associations with sensorimotor and limbic circuitry.

We further examined whether the altered FC was associated with clinical severity. Correlation analyses revealed that higher HB scores were positively correlated with FC between the DCP and both the precentral and postcentral gyri, as well as between the DRP and the fusiform gyrus, postcentral gyrus, and SMA. In contrast, FC between the DC and cerebellum Crus I showed a negative correlation with HB scores, while other DC-cerebellar connections were not significant.

These results suggest that striatal connectivity with sensorimotor regions reflects, to some extent, the degree of facial motor impairment. Although these correlations cannot establish causality, they support the functional relevance of the observed FC changes in the context of early facial nerve recovery.

Taken together, these findings highlight that both cortical and striatal circuits undergo dynamic reorganization during the early treatment of BP and that these neural changes are closely related to the degree of facial motor dysfunction. While the absence of a control group limits causal interpretation, the convergent alterations in ReHo, fALFFs, corticostriatal connectivity, and their associations with HB scores collectively suggest that acupuncture treatment may coincide with measurable neural adjustments within sensorimotor and striatal pathways. Future randomized controlled studies with larger samples will be essential to further clarify the mechanisms underlying these changes and to determine the specific contribution of acupuncture to facial nerve recovery.

## 5 Limitations

There are several limitations to this study that should be acknowledged. First, the absence of a control group (e.g., sham acupuncture) limits our ability to attribute the observed neural changes specifically to acupuncture rather than spontaneous recovery. Second, the sample size was relatively small, which may reduce statistical power and limit the generalizability of the findings. Importantly, based on these preliminary findings, we plan to conduct a larger, randomized controlled trial to more rigorously assess the causal contribution of acupuncture to neural and clinical recovery in BP.

## 6 Conclusion

Our results suggest that (1) the striatum is involved in the recovery process of peripheral nerve injury with acupuncture treatment, and (2) the dorsal regions of the striatum (DCP, DRP, and DC) may play a key role in peripheral nerve recovery. The finding may help clarify the underlying mechanisms of acupuncture in BP. We plan to further investigate the mechanisms of acupuncture in future studies.

## Data Availability

The raw data supporting the conclusions of this article will be made available by the authors, without undue reservation.

## Footnotes

1
http://www.nitrc.org/projects/conn
